# Comparison of newborn hearing screening results between well babies and neonates admitted to the neonatal intensive care unit for more than 5 days: Analysis based on the national database in Korea for 9 years

**DOI:** 10.1371/journal.pone.0235019

**Published:** 2020-06-19

**Authors:** Jiwon Chang, Seung-Ha Oh, Su-Kyoung Park

**Affiliations:** 1 Department of Otorhinolaryngology-Head and Neck Surgery, Kangnam Sacred Heart Hospital, Hallym University College of Medicine, Seoul, Korea; 2 Department of Otorhinolaryngology-Head and Neck Surgery, Seoul National University College of Medicine, Seoul, Korea; University of Alberta, CANADA

## Abstract

**Objective:**

The purpose of this cohort study is to compare newborn hearing screening (NHS) results between healthy newborns and neonates who were admitted to the neonate intensive care unit (NICU) for more than 5 days based on the national database for 9 years. Ultimately, we’ve tried to analyze the associated factors necessary to manage the national NHS program according to the group, which would help to establish policy to effectively detect and support hearing impaired children and which would help to control qualities.

**Methods:**

The Ministry of Health and Welfare (MHW) introduced a nationwide coupon-mediated program for the low-income class since 2009. The coupon consisted of two parts, the screening part and the confirming parts with the same unique number, and the MHW supported the cost of one screening test and one diagnostic auditory brainstem response (ABR) test for infants who did not pass from the screening test. We have analyzed the screening test performing rate, the referral rate according to the screening methods or institutions, the prevalence of hearing loss, and the average age of hearing loss diagnosis. Hearing loss was defined as any hearing impairment either unilateral or bilateral with the hearing threshold ≥ 40 dB nHL on the diagnostic ABR test, irrespective of its etiology.

**Results:**

A total of 524,371 newborns were enrolled in the study, and 506,634 (96.6%) neonates were in the “well-baby group (WBG)”, while 17,737 (3.4%) were in the “high-risk group (HRG)”. The referral rate of the screening test was 1.5% in average, 1.3% in the WBG, and 7.5% in the HRG. The referral rates varied according to the screening methods and screening institutions. The adjusted prevalence of HL was 5.6/1,000 in average, 4.6/1,000 in the WBC, and 28.8/1,000 in the HRG. The screening tests were performed 4.3 ± 6.7 days after birth and the diagnostic tests were done 62.7 ± 37.5 days after birth in WBG. In HRG, dates were 17.7 ± 19.3 days and 97.6 ± 51.4 days, respectively.

**Conclusions:**

The prevalence of hearing loss in infants who were hospitalized in NICU for more than 5 days was about seven times higher than that in healthy newborns. However, different referral rates were noted depending on both institutions and the screening methods. These differences need to be addressed in order to improve our program and ensure that all neonates with hearing loss, especially neonates with high risk factor, are detected and appropriately referred for the treatment.

## Introduction

Hearing is important for the development of language and communication skills [1, 2]. Since the incidence of the severe hearing loss (HL) in neonate is reported to be 1 to 2 per 1,000 newborns [2–4], an early detection of hearing loss and appropriate hearing rehabilitation are mandatory for both individuals and societies. Since the 1990s, newborn hearing screening (NHS) has been successfully performed using an automated auditory brainstem response (AABR) or an otoacoustic emissions screening test [2, 4]. The Joint Committee on Infant Hearing (JCIH) has recommended the ‘1-3-6’ guidelines for the early detection and intervention (EHDI) of HL; all newborns should be screened for hearing by 1 month of age, all infants who do not pass screening test should get a diagnostic audiological evaluation by 3 months of age, and infants who are confirmed with HL should start an appropriate intervention by 6 months of age [2, 4].

In South Korea, the government recognized the significance of NHS and had initiated NHS pilot programs since 2007, and the Ministry of Health and Welfare (MHW) started a nationwide coupon-mediated NHS program through 2009 to 2018, primarily for newborns of low-income families. Newborns who were born in the households with a median income of 72% or less, which included both all low-income class (less than 50% of median income) and some of the middle-income class (from 50% to 72% of median income) were supported with the nationwide coupon-mediated NHS program in these periods [5]. An NHS coupon consisted of a screening test part and a confirmation test part, and the government financially supported the cost of the first NHS test and one diagnostic auditory brainstem response (ABR) test for infants who did not pass in the NHS tests. During these periods, newborns that were not included in low-income classes didn’t get the benefit from the government program but had to conduct all tests on their own expenses. After 11 years of conducting NHS pilot program (from 2007 to 2018), NHS test were finally covered by National Hearth Service starting from October 2018, so all neonates in Korea can perform hearing tests without economic burdens nowadays.

From 2010 and on, the nationwide coupon-mediated NHS program collected data from enrolled newborns and identified details whether they were admitted to NICU for more than 5 days, which is one of the high-risk factors for HL according to JCIH 2007. The referral rate is mentioned to be much higher in NICU neonates (2.8–9.2%) [6–9], and the incidence of congenital HL is reported to be 2 to 4/100 in NICU neonates which is much higher than 1.8 to 3/1000 in well-babies [10–12]. Accordingly, it is necessary to track and manage NHS in NICU neonates. However, there are few studies analyzing and comparing the NHS results of well-babies and NICU neonates especially with nationwide database.

So, the aims of this study were to compare NHS results between healthy neonates and neonates who were admitted to the NICU for more than 5 days based on the national database for 9 years, and to assess the referral rate according to the types of screening methods or institutions, the prevalence and severity of hearing loss, and the average age of HL diagnosis in both healthy and NICU neonates. Ultimately, we have tried to analyze the associated factors necessary to manage the national NHS program by separating the NICU and the healthy newborns, which would help to establish policy to detect and support children with hearing loss effectively and to control qualities of NHS program.

## Materials and methods

We analyzed the records of the national pilot newborn hearing screening (NHS) database from January 2010 through September 2018. In this period, a total 524,371 newborns were registered in this database. The national coupon-mediated pilot NHS project for mainly low-income families was implemented in 255 public health centers nationwide, and dozens of public health centers conducted the projects for all newborns in their area.

The coupon consisted of a screening part and a confirming part with a same unique number not related to personal information. The screening part contained the blanks to fill for the data of (1) the birth of neonate, (2) the type of screening clinic or hospital, (3) NHS performed date, (4) screening methods (either with AABR or (automated) otoacoustic emissions ((A)OAE), (5) screening test results, and (6) whether neonates were admitted to the NICU for more than five days. The coupon for the confirming part was for neonates who have not passed the NHS test and had to perform a follow-up diagnostic auditory brainstem response (ABR) test; it had blanks to fill in for (1) the birth of neonate, (2) results of NHS, (3) the type of hospital performing the diagnostic test, (4) the ABR date, and (5) the hearing threshold. The pregnant woman in the family received a NHS coupon at her residential public health center before childbirth, and when she gave birth, she submitted the coupon to designated NHS hospital for her newborn to undergo hearing screening test. Designated hospitals filled out the information on the screening coupon and submitted it to the MHW and charged for the test fee. The parents or caregivers of the newborns were instructed to visit the designated clinics or hospitals within 1 month of age for AABR or (A)OAE screening test. However, if the newborn stayed at the NICU for more than 5 days as a high-risk group, the screening tests were supported even after 1 month of age. The outcomes of NHS were presented as either “pass” or “refer”. The Ministry of Health and Welfare collected all national coupons and built a NHS database.

We categorized the all newborns into two groups, the ‘well-baby group (WBG)” who was born healthy or admitted to the NICU less than 5 days and the ‘high-risk group (HRG)’ who was hospitalized in NICU for more than 5 days. The number of newborns screened and referred, the prevalence of hearing loss and test performed dates after birth were investigated. Hearing impairment was defined as any hearing loss either unilateral or bilateral with the hearing threshold ≥ 40 dB nHL on the diagnostic ABR test, irrespective of its etiology.

The study protocol was approved by the institutional review board of Hallym University Kangnam Sacred Heart Hospital (IRB no. HKS201910020) and waved to inform the consent by the Institutional Review Board because of retrospective database study nature.

Statistical analyses were performed using SAS 9.2 Version (SAS Institute Inc., Cary, NC, USA). Statistical significance was set to *p* < 0.05. Statistical significance was assessed by Pearson chi-square or Fisher’s exact test, with Bonferroni correction of *p* values for multiple comparisons.

## Results

### Hearing screening performing rate and the referral rate

**Fig 1:**
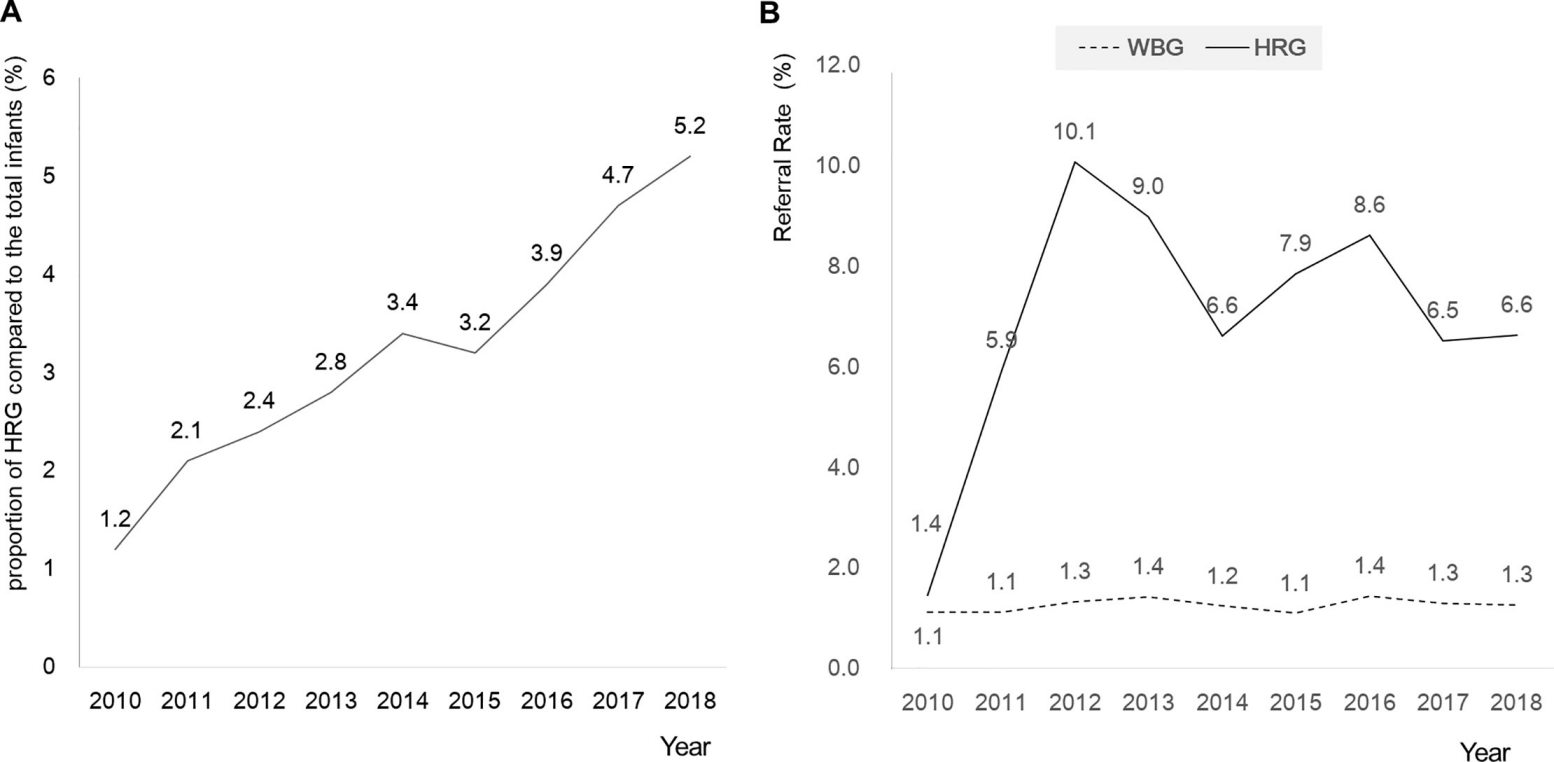
The annual proportion of the high-risk group (HRG) (A), and the annual referral rates of the well-baby group (WBG) and HRG (B) during 9 year-national pilot program. NHS: newborn hearing screening, well-baby group: newborns born healthy or stayed at the neonatal intensive care unit less than 5 days, High-risk group: infants who were hospitalized in the neonatal intensive care unit for more than 5 days.

A total of 524,371 newborns were enrolled in the study, and 506,634 (9.6%) neonates were in the WBG, while 17,737 (3.4%) were in the HRG (Table 1). During the 9-year NHS pilot program between years 2010 to 2018, the percentage of high-risk group increased annually (Fig 1A). The referral rate of the screening test was 1.3% in the WBG, 7.5% in the HRG and 1.5% in total (Table 1). The annual change of referral rate for both groups is shown in Fig 1B.

**Table 1:** Newborn hearing screening (NHS) state of well-baby group (WBG) and high-risk group (HRG) in NHS pilot program from 2010 to 2018 in Korea.

	WBG		HRG		Total		Missing value
	n	%	n	%	n	%	
Total NHS tests	506,634	96.6	17,737	3.4	524,371	100.0	44
Referred newborns,	6,436	1.3	1,327	7.5	7,698	1.5	0
By NHS method							
AABR	448,709	88.6	14,793	83.6	463,502	88.4	
(A)OAE	57,837	11.4	2,903	16.4	60,740	11.6	
Subtotal	506,546	100.0	17,696	100.0	524,242	100.0	129
By screening hospital or clinics							
Maternity Clinics	474,881	93.7	4,262	24.0	479,143	91.4	
ENT Dept. of General Hospitals	21,983	4.3	13,361	75.3	35,344	6.7	
Other Local Clinics	9,757	1.9	112	0.6	9,869	1.9	
Subtotal	506,621	100.0	17,735	100.0	524,356	100.0	15

Total number of newborns who enrolled this study was 524,371

WBG: well-baby group of newborns born healthy or stayed at the neonatal intensive care unit (NICU) less than 5 days, HRG: high-risk group of neonates who were hospitalized in the NICU for more than 5 days, AABR: automated auditory brainstem response, (A)OAE: (automated) otoacoustic emissions, ENT: ear, nose, and throat, ABR: auditory brainstem response, AABR: automated auditory brainstem response, (A)OAE: (automated) otoacoustic emissions, ENT: ear, nose, and throat

When we analyze the NHS results by the screening methods, WBG was screened with AABR in 448,709 cases (88.6%) and with (A)OAE in 57,837 cases (11.4%). In HRG, the screening was performed with AABR in 14,793 cases (83.6%) and with (A)OAE in 2,903 cases (16.4%). We’ve identified that AABR screening methods (88.4%) were preferred than (A)OAE methods (11.6%) during the 9-year NHS pilot program (Table 1). The referral rate among WBG was 1.3%, but when we analyzed the referral rate by screening methods, it was 1.1% by AABR methods and 2.9% by (A)OAE methods. Also, the average referral rate among HRG was 7.5%, but was 6.8% by AABR methods and 11.3% by (A)OAE methods (Table 2).

**Table 2:** Referral state from newborn hearing screening (NHS) according to NHS methods from 2010 to 2018 in Korea.

	WBG			HRG		
NHS methods	Number of NHS test	Number referred	Referral rate (%)	Number of NHS test	Number referred	Referral rate (%)
AABR	448,709	4,757	1.1	14,793	1,000	6.8
(A)OAE	57,837	1,679	2.9	2,903	327	11.3
Total	506,546	6,436	1.3	17,696	1,327	7.5

Number of missing infants was approximately 129 when we analyzed by NHS methods.

WBG: well-baby group of newborns born healthy or stayed at the neonatal intensive care unit (NICU) less than 5 days, HRG: high-risk group of neonates who were hospitalized in the NICU for more than 5 days, AABR: automated auditory brainstem response, (A)OAE: (automated) otoacoustic emissions

**Fig 2:**
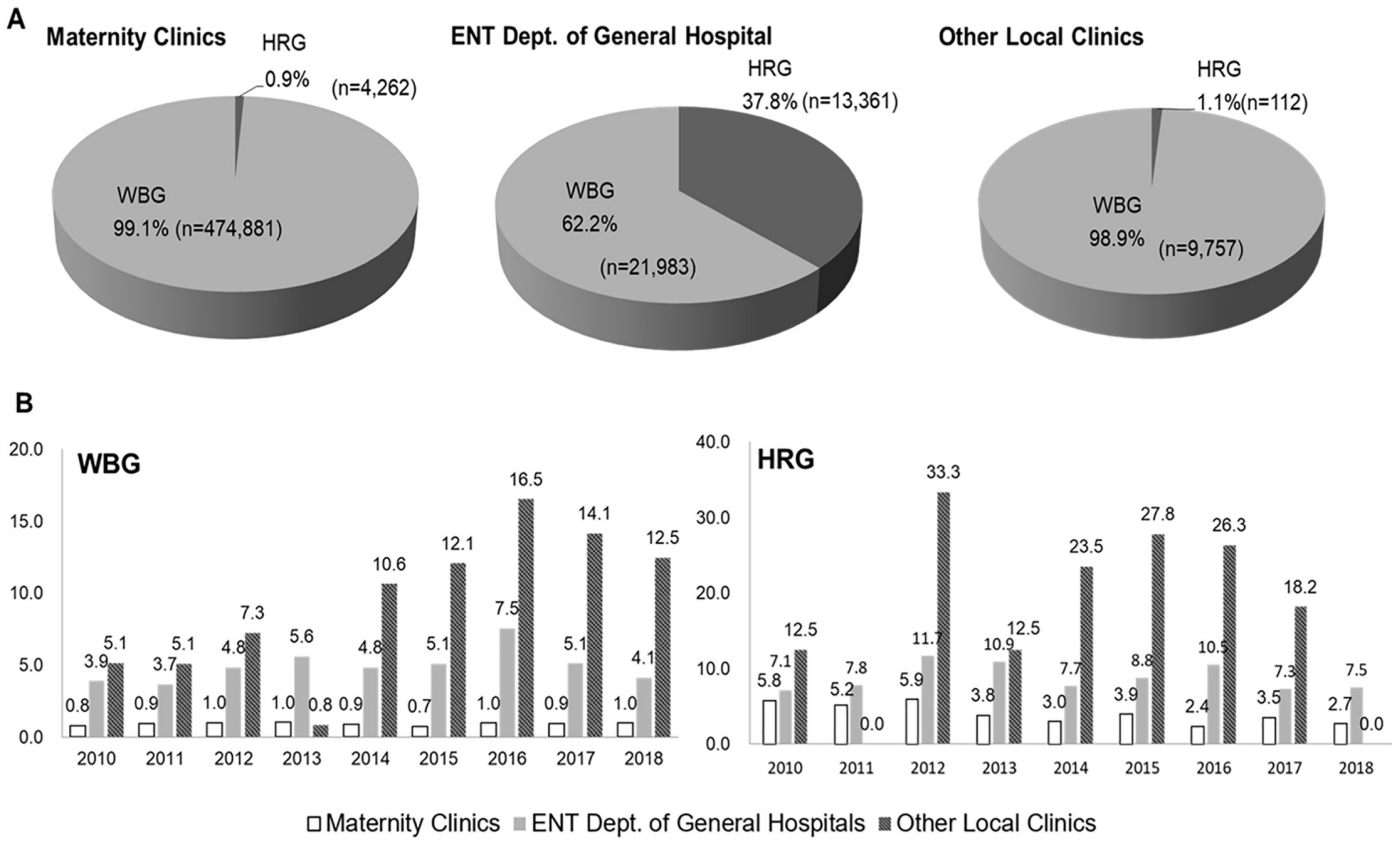
Status of newborn hearing screening in WBG and HRG according to screening hospitals. A. The percentage of WBG and HRG according to screening hospitals. The proportion of HRG was significantly different among three screening clinics and hospitals (*p*<0.001; Pearson’s chi-square test with Bonfferoni correction of 0.017 significance). B. Annual referral rates of WBG and HRG according to screening hospitals. The overall referral rates were significantly higher in the order of other local clinics, ENT department of general hospital, and maternity clinics in both (*p* <0.001). WBG: well-baby group of newborns born healthy or stayed at neonatal intensive care unit (NICU) less than 5 days, HRG: high-risk group of neonates who were hospitalized in the NICU for more than 5 days, ENT: ear, nose, and throat.

Then we analyzed the NHS results by the screening hospitals. WBGs were distributed with different percentages among hospitals; 93.7% (n = 474,881) were screened in the maternity clinics, 4.3% (n = 21,983) were screened in the ENT department of general hospitals and 1.9% (n = 9,757) were screened in various local clinics (Table 1). HRGs were distributed as 24% (n = 4,262) in the maternity clinics, 75.3% (n = 13,361) in the ENT department of general hospitals and 0.6% (n = 112) in various local clinics (Table 1). Maternity clinics and other local clinics performed the NHS for the WBG mostly (99% in both groups) but ENT department of general hospital had high proportion of HRG (38%) (Fig 2A). The proportion of HRG was significantly different among three different screening hospitals (*p*<0.0001). The referral rate was 0.9% for WBG and 3.6% for HRG in maternity clinics (Table 3). The referral rate was 5.1% for WBG and 8.6% for HRG in ENT departments in general hospitals. Moreover, the referral rate was 8.9% for WBG and 18.8% for HRG in other local clinics. The reported annual referral rates for WBG and HRG according to screening hospitals are shown in Fig 2B for the detail. The overall referral rates were higher in the order of other local clinics, ENT department of general hospital, and maternity clinics in both groups (*p* <0.0001)

**Table 3:** Referral state from newborn hearing screening (NHS) according to screening hospital from 2010 to 2018 in Korea.

	WBG			HRG		
Screening hospital	Number of NHS test	Number referred	Referral rate (%)	Number of NHS test	Number referred	Referral rate (%)
Maternity Clinics	474,881	4,370	0.9	4,262	153	3.6
ENT Dept. of General Hospitals	21,983	1,116	5.1	13,361	1,154	8.6
Other Local Clinics	9,757	868	8.9	112	21	18.8
Total	506,621	6,354	1.3	17,735	1,328	7.5

Number of missing infants was approximately 15 when we analyzed by screening hospitals.

WBG: well-baby group of newborns born healthy or stayed at neonatal intensive care unit (NICU) less than 5 days, HRG: high-risk group of neonates who were hospitalized in the NICU for more than 5 days, ENT: ear, nose, and throat.

### Prevalence of hearing loss

The total identified number of HL infants was 667 (537 in WBG, 117 in HRG) (Table 4). The WBG had 300 unilateral HL infants and 257 bilateral HL infants. The HRG had 50 unilateral HL infants and 67 bilateral HL infants. Then we’ve adjusted the simple prevalence of HL with mean documented ABR conducing rate which was 22.9%, and obtained the adjusted prevalence of HL. The adjusted prevalence of HL among total 524,371 infants was 0.56 for any either side HL, 0.30 for unilateral HL and 0.26 for bilateral HL. In the group of 506,634 WBG infants, the adjusted prevalence of HL was 0.46; 0.26 for unilateral and 0.20 for bilateral HL. In HRG, the adjusted prevalence of HL was 2.88; 1.23 for unilateral and 1.65 for bilateral HL (Table 4).

**Table 4:** Prevalence of hearing loss (HL) in the WBG and HRG in 9 years of newborn hearing screening pilot program in Korea.

	Number of infants enrolled	Number of infants with HL			Prevalence of HL (%)			Adjusted prevalence of HL with mean documented ABR conducting rate (%)		
		Unilateral	Bilateral	Total	Unilateral	Bilateral	Total	Unilateral	Bilateral	Total
WBG	506,634	300	257	537	0.06	0.05	0.11	0.26	0.20	0.46
HRG	17,737	50	67	117	0.28	0.38	0.66	1.23	1.65	2.88
Total	524,371	357	310	667	0.07	0.06	0.13	0.30	0.26	0.56

Mean documented auditory brainstem response (ABR) conducting rate of infants who did not pass screening test was22.9%.

WBG: well-baby group of newborns born healthy or stayed at the neonatal intensive care unit (NICU) less than 5 days, HRG: high-risk group of infants who were hospitalized in the NICU for more than 5 days

**Fig 3:**
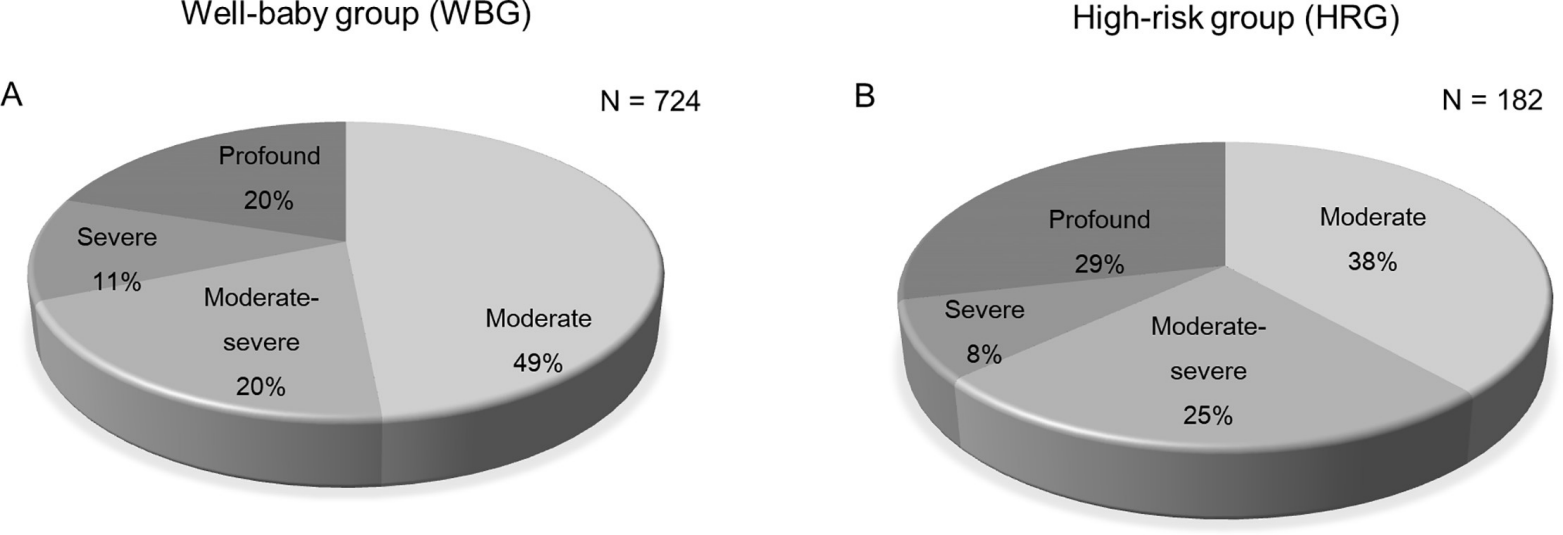
The distribution of hearing loss (HL) according to the degree of HL in WBG and HRG (N = ear). A. The proportion of ears with hearing impairment in WBG. Total of 724 ears had HL; 351 (49%) ears had moderate HL, 146 (20%) ears had moderate-severe HL, 82 (11%) ears had severe HL and 145 (20%) ears had profound HL. B. The proportion of ears with hearing impairment in HRG. Total of 182 ears had hearing impairment; 70(38%) ears had moderate HL, 45(25%) ears had moderate-severe HL, 15(8%) ears had severe HL and 52 (29%) ears had profound HL. The degree of HL was not significantly different between WBG and HRG (*p* = 0.6025). The 40–55 dB nHL was classified as moderate HL, 56–70 dB nHL as moderate to severe HL, 71–89 dB nHL as severe HL, and 90 dB nHL or higher as profound HL.

Then we analyzed the distribution of HL severity by the ear. We defined 40–55 dB nHL as moderate HL, 56–69 dB nHL as moderate to severe HL, 70–89 dB nHL as severe HL, and 90 dB nHL or higher as profound HL. In WBG, total of 724 ears had hearing loss; 351(49%) ears had moderate HL, 146(20%) ears had moderate-severe HL, 82(11%) ears had severe HL and 145 (20%) ears had profound HL (Fig 3A). In HRG, total of 182 ears had hearing loss; 70(38%) ears had moderate HL, 45(25%) ears had moderate-severe HL, 15(8%) ears had severe HL and 52 (29%) ears had profound HL (Fig 3B). The degree of HL was not significantly different between WBG and HRG (*p* = 0.6025)

### Performed dates of hearing screening test and the diagnostic test

**Fig 4:**
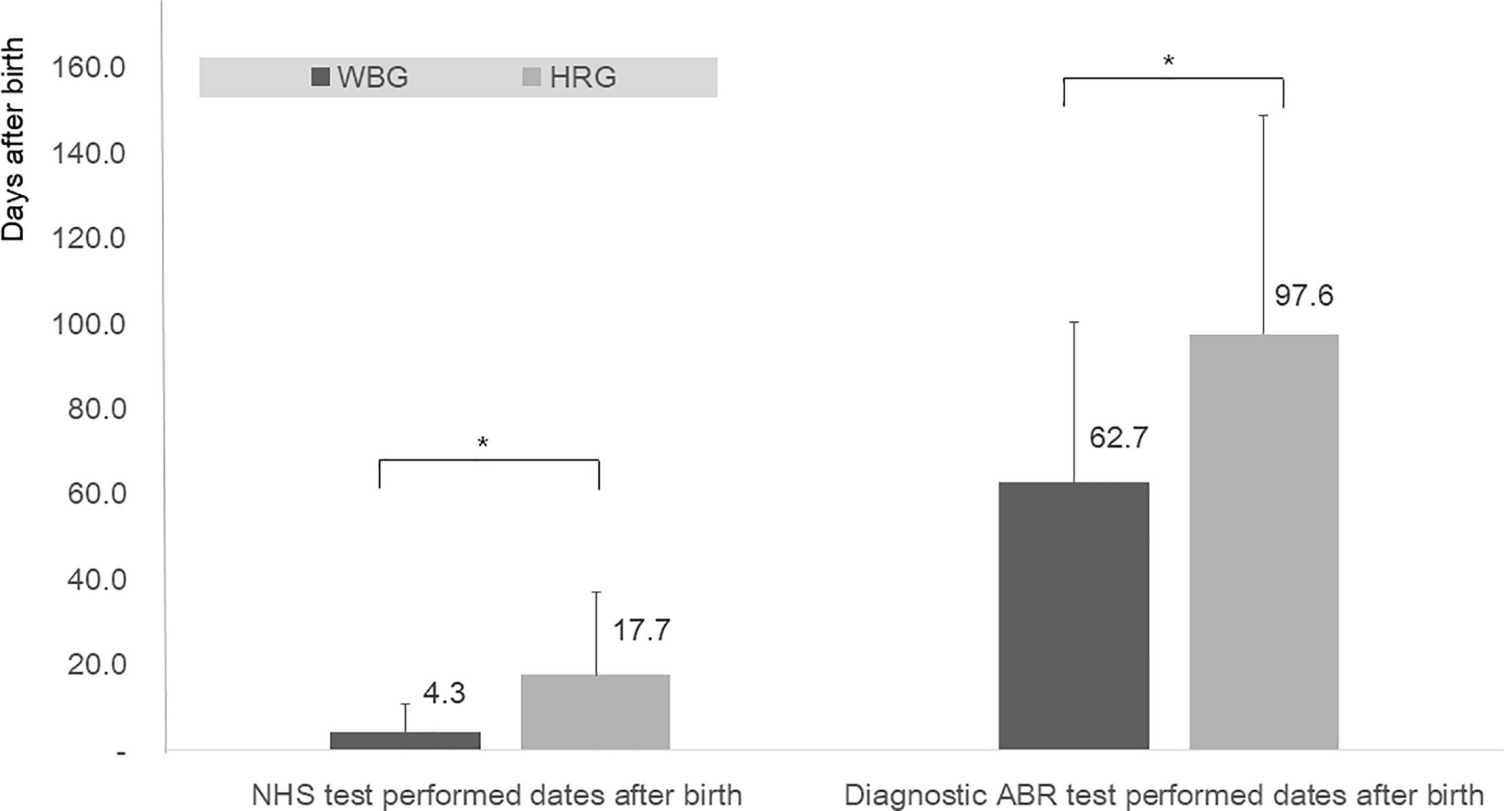
Performed dates of hearing screening test (A) and the diagnostic test (B) in well-baby group (WBG) and high-risk group (HRG). In WGB, the average screening tests were performed 4.3 ± 6.7 days after birth and the referred infants were diagnosed with ABR 62.7 ± 37.5 days after birth. In HRG, the average screening tests were performed 17.7 ± 19.3 days after birth and the referred infants were diagnosed with ABR 97.6 ± 51.4 days after birth. In the WBG, both screening and confirmatory tests were performed significantly earlier than those of HRG. *: *p<*0.05 (*p<*0.0001 in screening test and *p* = 0.0102 in diagnostic test, Wilcoxon rank sum test).

In WGB, the average screening tests were performed 4.3 ± 6.7 days after birth and the referred infants were diagnosed with ABR 62.7 ± 37.5 days after birth. In HRG, the average screening tests were performed 17.7 ± 19.3 days after birth and the referred infants were diagnosed with ABR 97.6 ± 51.4 days after birth. The screening test dates were significantly longer in HRG (*p*<0.0001). The diagnostic test dates were also significantly longer in HRG (*p* = 0.0102) (Fig 4).

## Discussion

There have been studies comparing NICU and healthy neonates in several countries and reporting associated risk factors. But few studies have been conducted in a large population with national database as this study, and a total of 524,371 newborns (WBG with 506,634 newborn and HRG who were admitted in NICU more than 5 days with 17,737 newborns) was enrolled [9, 13–15]. The purpose of this study was to compare NHS results between healthy newborns and infants who were admitted to the NICU for more than 5 days, to analyze the associated factors necessary to manage the national NHS program and to establish effective policy to support hearing impaired children. Among total of 524,371 newborns in our study, 96.6% (506,634) were WBG, and 3.4% (17,737) were HRG. The mean proportion of HRG increased annually. The elevated proportion of HRG in Korea can be based on the elevation of high-risk pregnancy due to the low birth rate, the increased average age of marriage, and high maternal age [16]. Also, an urban concentration has produced delivery vulnerable areas which led to the elevation of HRG percentage [17]. The increase in the incidence of HRG is positively related with the high incidence of hearing loss in HRG. Therefore, it is necessary to have a national-wide management of the HRG, and to establish database and tracking system for the HRG.

The mean referral rate of total enrolled newborns was 1.5% but the annual referral rate was not even in HRG in our study (Fig 1B). The inconsistency in the annual referral rate of HRG might be due to the lack of publicity or lack of knowledge regarding the designation methods at the beginning of the 9-year nationwide coupon-mediated NHS program. The WBG was supported for the screening test if the tests were done within a month after the birth, but the HRG were supported according to the adjusted chronologic ages because of possible immature and unhealthy state. Subsequently, if the caregivers or community centers did not recognize that the premature neonates could be supported by the national NHS program although tests were done after one month, they might not have applied for the support and their results could have been omitted from the database.

When we identified the referral rate according to the group, it was 1.3% for WBG and 7.5% for HRG. Although a referral rate of less than 4% for overall newborns is recommended for NHS program quality control such as JCIH and other guideline [2, 18–20], the reported referral rate is mentioned to be much higher in NICU neonates as 2.8–9.2% [6–9]. It is known that the referral rate elevates with increased incidence of hearing loss [6, 9], and NICU neonates exhibit a high incidence of hearing loss, at a rate 10 times greater than that of well-babies [12, 18]. However, in our study, the referral rate of 1.3% in WBG was rather low. It turned out in the unpublished survey performed by the MHW in 2009 that the maternity clinics had misconceptions in the beginning of the NHS program, misunderstood the screening tests for a confirmation test and performed the screening tests more than 10 times repeatedly. Consequently, there was a large effort for several years to correct the misunderstanding regarding NHS and to increase the investigator proficiency by several projects such as annual off-line NHS workshops, an online NHS training site development (2013) and the publication of NHS guidelines and position statement (2010 and 2018) [20]. Subsequently, all neonates were supported by National Hearth Service to conduct NHS starting from October 2018. That is, new NHS system started in 2018 allowed all newborns to be screened for the hearing and supported the cost of screening test. However, it did not provide the follow-up management which has been partly implemented with the previous coupon-mediated NHS program. Therefore, it is mandatory to develop uniform national registries and databases that incorporate standardized methodologies, obtain the results of hearing screening and diagnostic test, analyze and interpret the data, and could be used to guide for hearing rehabilitation for each individual [2, 4].

In this study, the referral rate differed with the screening methods and screening hospitals. The NHS tests were performed with AABR in 88.4% and (A)AOE in 11.6%. Also, NHS tests were conducted mostly in maternity clinics (91.4%) followed by ENT department of general hospitals (6.7%) and other local clinics (1.9%). The distinct characteristic of Korea is that the hearing screening tests were started by maternity clinics in the beginning and AABR devices were widely spread by instrument corporations in 1990s. However, since there was lack of a quality control, ENT departments in general hospitals and university hospitals began to educate hearing specialists by offline workshops from early 2000, published guidelines and position statements (2010 and 2018); and these endeavors initiated a national NHS pilot program since 2007 [20]. However, as we have identified from the analysis, the referral rate obtained from maternity clinics during 9 years of program was 0.9% for WBG and 3.6% for HRG which is quite low. The quality control of NHS in maternity clinics is still ongoing topics to be evaluated and corrected. On the other hand, the NHS for HRG was mostly performed in ENT department of general hospitals which have NICUs, indicating that local clinics would have high referral rate due to low test numbers and the inexperience of investigators. It implies that to be successful in EHDI, the quality control should be implemented according to the type of screening clinics and hospitals.

In a systemic review of 53 articles, the average dropout rate which is loss to follow up after screening test was 20% in single-center studies and 21% in multiple-center studies. Reasons of a high dropout rate were the educational disparity, lack of adequate knowledge of parents, and lack of an adequate data management system which is the most important strategy to decrease the dropout rates during follow-up period after screening [21]. If we consider infants who are not included in the documented diagnostic ABR conducting rate, the number of hearing-impaired infants may be higher than reported data. In our study, the simple prevalence of hearing loss was 1.1/1,000 for WBG, 6.6/1,000 for HRG and 1.3/1,000 for total enrolled neonates. However, when we adjusted the prevalence of HL with the mean documented ABR conducting rate which was 22.9%, the adjusted prevalence of HL was 5.6/1,000, 4.6 /1,000 for WBG and 28.8 /1,000 for HRG.

When we analyzed the distribution of hearing loss severity by the ear, WBG had high proportion of moderate hearing loss (49%) and had 31% of severe or profound hearing loss which would require surgical interventions. Meanwhile, HRG had less percentage of moderate HL (38%) and higher proportion of moderate-severe, severe and profound hearing loss, but there was no significant difference between two groups. We should consider the possibility of middle ear effusion and resolution of the middle ear status for moderate degree HL, since otitis media with effusion is a common cause of failed infant hearing screening test [2, 22].

The JCIH has recommended the ‘1-3-6’ guidelines for the EHDI for children who have deaf or hard of hearing [2, 4]. In our study, WBG performed the screening test by 4.3 days after birth, the diagnostic test by 62.7 days after birth and the results were in accord with the EHDI guidelines. However, HRG figures were higher than those of WBG; the screening test was performed by 17.7 days after birth, the diagnostic test by 97.6 days after birth. Nevertheless, when we consider the adjusted chronologic age in the HRG, the tests are supposed to be conducted timely, also coincide with the EHDI guidelines.

There are some factors or limitations and need-improvements in our national NHS program. First, the diagnostic ABR performing rates should be increased. Second, as mentioned above, it is mandatory to have a quality control tailored to the type of screening hospitals. Third, it is required to adopt AABR as one of screening methods in HRG. Fourth, national NHS program should focus on the hearing rehabilitation of individuals through national registries and database. Finally, HRG should be managed and tracked separately, because they have higher chances of delayed hearing loss or auditory neuropathy. That is, to detect the false negative, delayed or progressive hearing loss, it is necessary to perform audiologic evaluations and continuous surveillances and management of the HRG.

## Conclusions

The prevalence of hearing loss in infants who were hospitalized in NICU for more than 5 days was about seven times higher than that in healthy newborns. However, different referral rates were noted depending on both institutions and the screening methods. These differences need to be addressed in order to improve our program and ensure that all neonates with hearing loss, especially neonates with high risk factor, are detected and appropriately referred for the treatment.

## Supporting information

S1 Checklist Strobe statement—checklist of items that should be included in reports of *cohort studies*. (DOC)
